# A Canonical Theory of Dynamic Decision-Making

**DOI:** 10.3389/fpsyg.2013.00150

**Published:** 2013-04-02

**Authors:** John Fox, Richard P. Cooper, David W. Glasspool

**Affiliations:** ^1^Oxford UniversityOxford, UK; ^2^University College LondonLondon, UK; ^3^Birkbeck, University of LondonLondon, UK; ^4^Deontics ResearchOxford, UK

**Keywords:** decision-making, autonomous agents, clinical decision-making, unified theories of cognition, cognitive systems

## Abstract

Decision-making behavior is studied in many very different fields, from medicine and economics to psychology and neuroscience, with major contributions from mathematics and statistics, computer science, AI, and other technical disciplines. However the conceptualization of what decision-making is and methods for studying it vary greatly and this has resulted in fragmentation of the field. A theory that can accommodate various perspectives may facilitate interdisciplinary working. We present such a theory in which decision-making is articulated as a set of canonical functions that are sufficiently general to accommodate diverse viewpoints, yet sufficiently precise that they can be instantiated in different ways for specific theoretical or practical purposes. The canons cover the whole decision cycle, from the framing of a decision based on the goals, beliefs, and background knowledge of the decision-maker to the formulation of decision options, establishing preferences over them, and making commitments. Commitments can lead to the initiation of new decisions and any step in the cycle can incorporate reasoning about previous decisions and the rationales for them, and lead to revising or abandoning existing commitments. The theory situates decision-making with respect to other high-level cognitive capabilities like problem solving, planning, and collaborative decision-making. The canonical approach is assessed in three domains: cognitive and neuropsychology, artificial intelligence, and decision engineering.

## Introduction

The ability to respond flexibly to changing circumstances is fundamental to adaptive behavior in humans and other animals, and to artificial systems such as autonomous software agents and robots. Decision-making is a major source of theoretical questions (e.g., in economics, cognitive and social psychology, computer science, and AI) and practical challenges (e.g., in business, politics and conflict management, investments and insurance, voter and consumer behavior, law, and medicine). This vast range of interests has unfortunately led to great divergence of research methodologies (e.g., empirical observation, mathematical analysis, computational modeling, philosophical discourse) and fragmentation of decision research. There have of course been major attempts to develop domain independent perspectives, such as normative frameworks (e.g., Bayesian and expected utility models; game theory), behavioral decision models (e.g., heuristics and biases and prospect theory), and information processing approaches (e.g., neural networks and cognitive architectures). However, these attempts tend to take place from the viewpoint of one community and opportunities for sharing insights and theoretical unification are missed.

We offer a unified view of decision-making which addresses the following questions.

1. How can we understand the dynamic lifecycle of decision-making: from the situations and events that make a decision necessary to the influence of prior knowledge, beliefs, and goals which determine how a decision will be framed, preferences arrived at, and commitments to actions made (Fox and Das, 2000)?
2. What are the *general functions* that underpin and constrain the processes that implement such a lifecycle for any kind of cognitive agent, whether the agent is natural or artificial?
3. How does decision-making, conceived in this very general way, fit within cognitive science’s strategic objective of a *unified theory of cognition* that can cut across psychology, computer science, AI, and neuroscience (e.g., Newell, 1990; Anderson, 2007; Shallice and Cooper, 2011)?
4. How can we apply this understanding to *decision engineering*, drawing on insights into how decisions are and/or ought to be made to inform the design of autonomous cognitive agents and decision support systems (e.g., Fox et al., 2003; Fox et al., 2010)?

The goal of a unified theory is ambitious, some will say hubristically so. However despite the long-term objective of unification the objective of this paper is more modest: to establish a framework and a language which can facilitate discussion between decision researchers in different communities, from theorists with distinct but complementary perspectives to practitioners such as doctors, engineers, and managers who wish to improve their decision-making.

We begin with a brief overview of classical approaches to decision theory, which we contrast with theories of dynamic decision-making (DDM). We then introduce some perspectives on DDM which we believe have been neglected. This paves the way for the introduction of the canonical framework, in which we seek to understand DDM in terms of a number of key capabilities which, we assert, a cognitive agent must have. These *canons* are abstracted from field-specific details; we acknowledge there are countless possible implementations and interpretations of the canons. In the final section we assess the canonical theory in three restricted settings: cognitive neuropsychology; artificial intelligence; and the design of practical decision support systems.

## Traditional Methodologies and Theories in the Decision Sciences

Decision-making may be defined in very general terms as a process or set of processes that results in the selection of one item from a number of possible alternatives. Within this general definition, processes might be natural and conscious, as in deliberate choice amongst alternatives, but also unconscious (as in selecting the grip to use when grasping an object) or artificial (as in an expert system offering decision support). Moreover, decisions can be about *what to do* (*action*), but also about *what to believe* (*opinion*). We will later extend this definition to cover DDM, but for now it is sufficient to summarize three classical perspectives that are common in decision research.

### Prescriptive theories of rational choice

Prescriptive decision theories have emerged from mathematics and mathematical economics where *rational choice* is taken to be central to understanding economic behavior and managing economic systems efficiently. The methodology focuses on establishing rational axioms for making decisions under uncertainty and consequences for systems of trade and commerce against defined valuations. The axioms typically express mathematical constraints which, if violated, can lead a decision-maker into suboptimal choices. Such prescriptive theories tend to be agnostic about the processes or algorithms that might implement or operationalize the mathematical constraints. Despite their theoretical importance the application of classical prescriptive decision models suffers from the practical problem that it is often difficult to estimate the quantitative parameters that they require (e.g., probabilities, utilities). Although they have informed research on human decision processes they provide limited insight into them and ignore key theoretical problems in DDM.

### Descriptive theories of natural decision-making

The goals of psychology are to explain human behavior and predict performance, irrespective of how performance compares with rational norms. Early psychological models of decision-making were influenced by rationalist theories as sources of theoretical concepts and normative standards against which to assess human decision-making, but there has been a trend away from this in recent decades. For example Simon’s (1957) concept of “bounded rationality” emphasized human limited information processing capacity and strategies for accommodating this (e.g., satisficing). Kahneman and Tversky’s heuristics and biases program also sought a more realistic account of cognitive processes in decision-making (Tversky and Kahneman, 1974) and Kahneman and Tversky (1979) developed a better description of how people evaluate potential losses and gains compared to mathematically prescribed norms. More recently Gigerenzer and Todd (2000) argue for the practical importance of simple heuristic strategies for fast decision-making.

### Design frameworks for decision engineering

In contrast to the above perspectives, designers of *decision support systems* and other decision-making software view decision processes and applications in a way that is analogous to designing objects like bridges and buildings. Decision engineers therefore tend to be interdisciplinary in their approach, exploiting mathematical and normative theories, or being inspired by human decision-making as inartificial neural networks and “expert systems,” or adopting a pragmatic mix of both. Decision engineers’ primary concerns are with achieving specific objectives and they may adopt any methods that are effective in achieving this goal. Despite considerable practical success decision engineering risks use of *ad hoc* rather than principled design theories and, as a consequence, there can be considerable uncertainty about the performance of decision systems in practice.

## Dynamic Decision-Making

In all the above perspectives decision-making is typically viewed as a choice between a set of *predefined options*. This is unsatisfactory because a decision typically arises within a wider context, in which it is necessary to recognize when a situation or event requires a decision, determine the set of options, establish criteria for determining preferences, resolve conflicts, and so on. This is the focus of DDM.

Edwards (1962) characterized DDM in terms of the following features: (1) a series of decisions is required to achieve a goal; (2) decisions are not independent (decisions are constrained by earlier decisions); (3) the state of the problem changes, with changes in the environment, or as a consequence of the decision-maker’s actions, and (4) decisions are made in real time. DDM is fundamental in practical domains, such as fire fighting, factory production, clinical decision-making, air traffic control, military command, and control, emergency management. In this section we briefly overview a few attempts to address the complexity of DDM.

### Naturalistic decision models

Writing for practitioners Drummond (1991) provides a “synoptic model” of a full decision cycle as follows:

1. Identify problem
2. Clarify and prioritize goals
3. Generate options
4. Evaluate options
5. Compare predicted consequences of each option with goals
6. Choose option with consequences most closely matching goals

She also identifies features of practical decision-making that are not so much to do with the dynamics of choice but are significant for a general account of decision-making, including Individual differences; collaboration, and joint decision-making; multiple criteria and conflicting goals, and “problems within problems” (any step in a decision process can unexpectedly turn into a new problem as complex as the original one).

Klein’s (2008) Naturalistic Decision-Making program seeks to understand how people make decisions in the real world and how they perform tasks in demanding situations. Important real world challenges include vagueness of goals, high stakes, significant levels of uncertainty about what is the case or what the consequences of actions might be, time pressure, team and organizational constraints, changing circumstances, and limited knowledge and experience. From extensive studies of experts such as fire fighters, he concluded that “by 1989, it was fairly clear how people didn’t make decisions. They didn’t generate alternative options and compare them on the same set of evaluation dimensions. They did not generate probability and utility estimates for different courses of action” (2008, p. 456). Klein wants to go beyond the simple choice paradigm of classical decision theory to ask how people maintain situational awareness, make sense of events, formulate goals, and construct plans to achieve them.

Brehmer (1992) also advocates a naturalistic approach, adopting computer simulations of real world situations such as fighting a forest fire as a research platform. Here it is possible to investigate features of human performance like the development and use of decision strategies, the importance of feedback, and how people learn to control complex and evolving situations, which find little place in classical decision theory or laboratory experiments. Since the contingencies of the simulation are under complete program control even complex decision-making can be studied in a systematic way.

In a well known discussion of human expertise Shanteau (1987) observed that expert decision-makers have many capabilities that cannot be accounted for with traditional theories. They know what to attend to in busy environments, what is *relevant* to decisions, and when to make exceptions to general rules, adapt to changing task conditions, and find novel solutions to problems. They also know a lot about what they know and can articulate the rationale for their decisions in terms of the relevant evidence and facts that support different options.

A general theory of DDM must address such capabilities, whether the focus is rational choice, human cognition, or engineering.

### Cognitive architectures

As early as the 1950s Newell and Simon were exploring the value of computational concepts in understanding human cognition (e.g., Newell et al., 1958) which evolved into rule-based models (Newell and Simon, 1972) and finally the Soar project (Laird et al., 1987). Soar is relevant here because it was developed as a model of general intelligence that subsumed decision-making as a key component, and was seen as offering a unifying view of human and artificial intelligence^1^.

**Figure 1:**
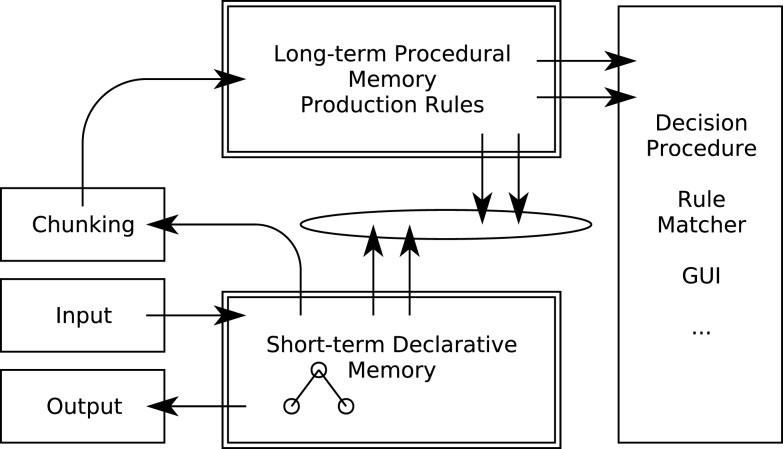
**The Soar information processing architecture**.

The Soar architecture (Figure 1) showed how a relatively simple information processing mechanism could carry out a wide range of cognitive tasks. It extended Newell and Simon’s established production rule approach by introducing some capabilities that a general theory of DDM needs to address, including dynamic generation of task goals, selection, and application of knowledge (rules) from long-term memory, and executing general problem solving strategies when no specific rules are available. In the latter case Soar “chunks” a new rule from the problem solving trace and adds it to long-term memory for use in future similar circumstances (Laird et al., 1987; Newell, 1990).

**Figure 2:**
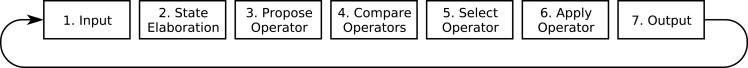
**The Soar dynamic decision cycle**.

A central mechanism of the Soar architecture is a cyclical *decision procedure* (Figure 2). This reacts to and interprets new data (“elaboration”) and makes a decision by comparing alternative cognitive operations based on the interpretation, selecting one and then applying it, resulting in a change in the state of short-term memory. This leads to a new cycle of processing.

Soar has been extensively used for modeling human performance on complex tasks and for designing and implementing expert systems, and is the foundation of Newell’s (1990).

Our own work has also focused on computational architectures for modeling DDM (Fox, 1980; Cooper et al., 2003) and high-level cognition using systematic methods and tools (Cooper and Fox, 1998; Cooper et al., 2002). We have compared several cognitive processing models, including: rule-based models; Bayesian inference and connectionist classifiers and heuristic architectures. The models were successful in that we were able to simulate behavior on complex decision tasks in some detail but firm theoretical conclusions have proved elusive, because:

1. Seemingly distinct theoretical accounts have comparable abilities to simulate observed behavior at similar levels of detail because, we believe, many competing theories have comparable descriptive and explanatory power. They may in fact be indistinguishable in principle. Debating which particular class of theory best describes human decision-making may be unproductive.
2. There was frequently more variance in individual subjects’ behavior than between the models. Even if there is a fixed decision-making mechanism (a la Soar) a decision-maker’s knowledge and what is learned on task are at least as important in explaining performance data. What a decision-maker “knows” will often have a greater impact on performance than any hypothetical rule-based, connectionist, Bayesian or other mechanism (cf. Newell, 1981).

### Artificial intelligence and autonomous agents

AI researchers have sought to design mechanisms for controlling robots and other automata in many DDM tasks, including situation monitoring and assessment, problem solving, scheduling, and planning through to cognitive vision and natural language understanding systems. Since the late 1980s there has been particular interest in the concept of *autonomous agents*, and in *multi-agent systems* in which agents cooperate to achieve shared goals.

In AI an autonomous agent is an entity (usually software) that inhabits some sort of environment and can react to situations and events and behave purposefully to achieve its goals. The environment may be physical (e.g., the agent is a robot or autonomous vehicle) or virtual (e.g., a simulation or the world wide web). Table 1 summarizes the main capabilities that agent theorists have sought to automate, under three headings: interaction with the environment, cognitive capabilities, and cognitive control.

**Table 1:** **Capabilities that are typical of agent systems described in the AI literature (Fox et al., 2003)**.

**INTERACTIONS WITH ENVIRONMENT**	
Perception	Observing and monitoring situations and events in the environment
Action	Executing actions that change or control the environment
Communication	Employing perception and action to interact with other agents
**COGNITIVE CAPABILITIES**	
Reasoning	Making inferences on the basis of environmental data, beliefs, goals, knowledge, etc.
Problem solving	Searching for explanations of observations, plans which will achieve goals etc.
Decision-making	Choosing between alternative hypotheses or actions
Scheduling	Sequencing actions and plans flexibly in response to circumstances
Planning	Constructing a set or sequence of actions to achieve a goal
Learning	Remembering solutions to newly encountered problems for future reuse
**CONTROL CAPABILITIES**	
Reactive behavior	Responding to situations and events in real time
Deliberative behavior	The application of cognitive capabilities in a purposive, coordinated way
Autonomy	Making plans, taking decisions, etc. without external programing or supervision

A few features of this table deserve comment. First, decision-making is an important cognitive function, but it is only one of a network of interrelated capabilities; reasoning; and problem solving can contribute to decision-making (in formulating decision options for example) while decision-making can contribute to problem solving and planning by assessing and selecting alternative problem solving strategies, plans, etc. Learning, in contrast, cuts across these capabilities in that any solution to a problem, plan or decision-making strategy that successfully achieves a goal may be worth remembering for future reuse. Second, autonomous decision-making can have multiple control regimes. Problem solving, planning, and even decision-making itself can be viewed as *deliberative* in that an agent reflects on its circumstances and goals to assemble one or more possible solutions to achieving its goals. On the other hand if the agent has learned from previous cases then it can operate *reactively* by retrieving candidate solutions from its knowledge base and making a decision by comparing the relative merits of the options.

A prominent computational theory of autonomous agent control and behavior draws on ideas from philosophy, psychology, and computer science in formalizing the concept of an agent. Following Bratman (1987) an agent is said to have *mental states* such as *beliefs*, *desires*, and *intentions* (Table 2). Such “BDI agents” have proved to be a practical basis for designing software agents (e.g., Rao and Georgeff, 1995). There are now many examples of agents which monitor their environments and maintain *beliefs* about them; generate goals (*desires*) with respect to the environment state, and if these are not consistent with their beliefs adopt plans (*intentions*) which will bring the environment into line with these goals.

**Table 2:** **Some of the mental/cognitive states that have been studied in AI**.

**COGNITIVE STATES**	
Beliefs	Specific information which an agent holds to be true at a particular moment in time
Desires	Specific goals which are currently influencing an agent’s behavior
Intentions	Specific commitments to actions or plans which an agent has decided to carry out
Knowledge	General theories, rules, functions etc as distinct from situation-specific beliefs, desires, and intentions

Knowledge, beliefs, desires, and intentions are often held to be mere “folk psychology,” of little scientific interest (e.g., Churchland, 1981). The fact that it has been possible to develop a formal interpretation of these and other cognitive states (e.g., Cohen and Levesque, 1990) and that they can be used to ground the design of practical software agents, suggests that such notions may have more theoretical power for understanding cognitive systems than is sometimes claimed. In the next section we discuss how they theories of mental states can illuminate our understanding of DDM.

## Mental States and Dynamic Decision-Making

**Figure 3:**
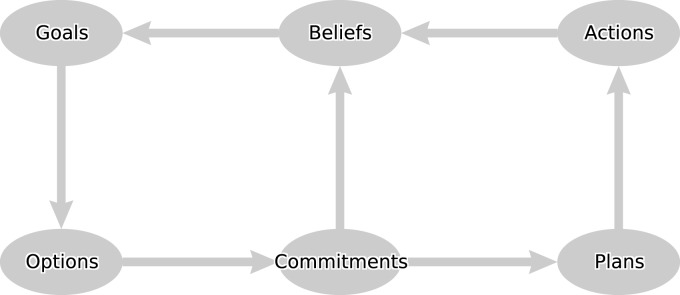
**The domino agent framework, an enhanced BDI agent model**.

Like Klein, Shanteau, Brehmer, and others our wish to understand high-level cognition has taken us out of the laboratory and into a world where decision-making is complex and indeed so difficult that even experienced practitioners, clinicians, make significant, and perhaps frequent errors^2^. We have studied decision-making in many routine medical settings including risk assessment; selection of tests and investigations; diagnosing the causes of a complaint; choosing treatments and prescribing drugs; implementing treatment plans; and team-based decision-making. This has led to a general framework for understanding clinical expertise and designing decision support tools that draws on some of the ideas discussed above. The “domino” model in Figure 3 is a computational architecture in which cognitive states provide an expressive and productive basis for describing cognitive processes throughout the decision cycle.

Each node of the domino represents a cognitive state of a particular type and each arrow represents a process for updating these states: beliefs can lead to new goals; goals to options for decision-making, and options can lead to commitments (about what to believe or what to do) with an associated rationale. We first explain the model by means of a simple medical scenario and then outline how the processes that generate the states can be computationally realized.

“Joan Smith has been rapidly losing weight, and there is no obvious reason for this.” In a clinical setting this scenario would typically lead to intentions to decide the cause of the weight loss and, if necessary, decide on the best action. There are several possible physical or psychological causes, and hence multiple hypotheses for explaining the patient’s complaint. Once the diagnostic options have been identified a decision-maker can determine what additional information to obtain (by asking questions, ordering investigations, etc.), and construct arguments for and against competing hypotheses based on the results. In due course the decision-maker can commit to a belief about the most convincing cause of the clinical problem.

Suppose the decision-maker accepts a diagnosis of gastric ulcer. This leads to a new goal: decide the best treatment for the ulcer. Knowledge of gastrointestinal disease suggests a range of treatment options, and arguments can be constructed for and against the alternatives based on efficacy, side-effects, costs, drug interactions, and so on. A decision about the most preferred treatment plan is based on an assessment of all the arguments. The preferred treatment may be simple, like prescribing a drug, or a complex care plan of many steps. Plan steps may lead to new observations, leading in turn to new goals and changes to the plan, and sometimes reversal of earlier decisions.

### Modeling what a decision-maker knows

Work in AI and cognitive modeling shows that an important challenge for decision theory concerns the representation and use of knowledge. Newell (1981) proposed that cognitive systems must be characterized at what he termed *the knowledge level* as well as the information processing level. This must describe the organization and semantics of knowledge, which enable and constrain cognitive processing. Since Newell’s paper there has been a great deal of work on modeling knowledge as frames and other knowledge representation techniques developed in AI, the roots of which are in the semantic networks and memory models developed in cognitive psychology, and more recently formalized as *ontologies*. We now give a brief overview of how ontological concepts are being used in computer science and AI to represent knowledge; this is informal and simplified presentation of technical work in this area but is a necessary foundation for the rest of the paper.

**Figure 4:**
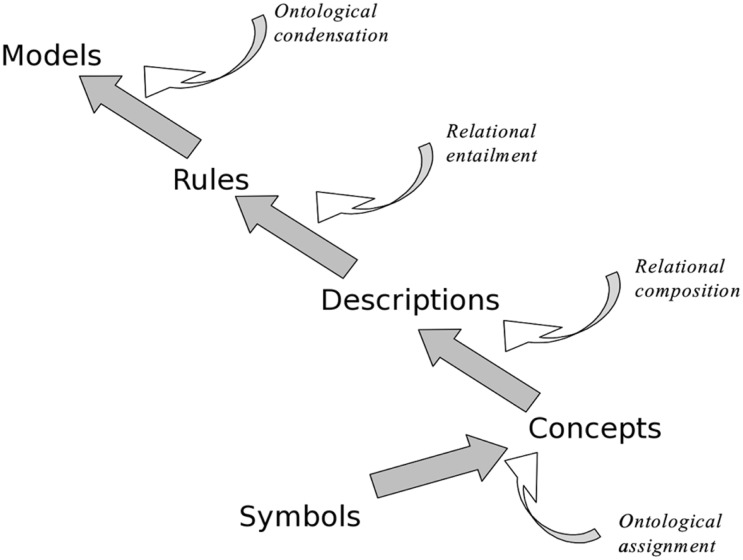
**The “ontological ladder,” which formalizes knowledge as a hierarchy of increasingly complex and semantically rich conceptual structures**.

An ontology can be thought of as a hierarchy of knowledge structures, in which each level of the hierarchy introduces a specific type of semantic information (Figure 4). For example the string “SCTID397825006” is a code for the medical term “gastric ulcer” in the SNOMED CT clinical coding system (Stearns et al., 2001). The code itself does not have any meaning; it is just a string of characters. A first level of meaning can be provided by an *ontological assignment*, linking the term “gastric ulcer” to a node in a *concept network* (as in “gastric ulcer” *is a kind of* “peptic ulcer”). This links the concept to more general categories through further assignments: “peptic ulcer” *is a kind of* “disease,” “disease” *is a kind of* “abnormal state,” and so on. The class structure facilitates an important form of reasoning called *inheritance*; the concept “gastric ulcer” can inherit properties from its super-class “peptic ulcer” and from the even more general class “disease” (e.g., every disease class and disease instance such as Joan Smith’s gastric ulcer inherits the property *has_symptoms*). Concepts are indicated here using quotes, and properties and relationships using italics.

Concepts can participate in other relations as well as *is a kind of* relations, such as *causal* relations (e.g., “gastric ulcer” *causes* “hematemesis”). If a patient has a gastric ulcer we may look for symptoms which are specifically caused by this disease (e.g., “hematemesis”) and, by inheritance, symptoms caused by more general kinds of peptic ulcer (e.g., “pain after meals”).

The concept “disease” is also semantically linked to other broad concepts, such as “treatments” which may be linked to diseases through relations like *controls*, *eradicates*, and so forth. Treatments also have subclasses in the ontology (such as “drugs,” “surgical treatments”) which have class-specific properties (e.g., *side-effects*, *method of administration*) as well as properties inherited from the general class (e.g., *effective for*, *cost of*).

Descriptions can be combined (e.g., “indigestion” is “present” *and* “patient” is “elderly”) and can participate in more complex structures like rules, as in

“indigestion” is “present” *implies* “possible diagnosis” is “peptic ulcer” “indigestion” is “present” *and* “patient” is “elderly” *implies* “possible diagnosis” is “gastric ulcer”

Finally descriptions and rules can be condensed into models. Two important kinds of model that are common in medicine are the Scenario and the Task which are the foci of much clinical discussion and decision-making. For example:

Scenario: “middle aged, overweight, male with hypertension not controlled by first-line therapy” Task: “eradicate tumor with surgery followed by 3 courses of adjuvant chemotherapy and annual follow-up for 3 yrs”

Models can participate in further ontological elaboration, forming elements of descriptions, rules, and higher-order models.

Ontologies are a major topic of research in knowledge representation, and currently offer the most sophisticated theoretical framework for understanding and applying knowledge in decision-making and other cognitive tasks.

## A Canonical Theory of Dynamic Decision Processes

The domino model was devised as an integrated theory of reasoning, decision-making, planning, and other capabilities that an autonomous agent may possess. In previous work we have interpreted each arrow in the model formally, as a specialized logic with a distinct set of non-classical axioms and inference rules (Das et al., 1997; Fox and Das, 2000). Although the model has been successfully used in many clinical applications it is clear that the functions modeled by the domino scheme could be understood in many other ways, and that different research communities would likely adopt different interpretations. Our aim here is to re-describe the framework in a more general way in which the arrows are viewed as *canonical functions* that can be instantiated in different ways to suit the purposes of different disciplines and traditions. Each function is first summarized informally, and then presented using a notational device called a *signature*. Signatures are commonly used to describe properties of computer programs in terms of their input-output constraints without specifying the internal details of how the function is to be implemented (e.g., Spivey, 1989)^3^. The level of abstraction provided by such signatures corresponds to a first approximation to a formal characterization of Marr’s computational level (Marr, 1982), specifying *what* is computed by the underlying process without specifying *how* it is computed (i.e., without specifying the algorithm that achieves the computation or the representations over which the algorithm operates).

### Canon 1: Belief maintenance

Any agent (natural or artificial) should maintain a consistent set of beliefs and expectations with respect to its current circumstances, updating these as its environment is observed to change.

Belief maintenance is fundamental to practical decision-making and is fundamental to all the decision models discussed here^4^. Beliefs need to be revised in the light of new observations, new beliefs, or new knowledge. There are countless proposals for how belief maintenance can/should be implemented; well known ones include probabilistic updating; fuzzy inference; classical deduction (propositional and predicate calculus), and non-classical logics (e.g., modal, abductive, inductive, and non-monotonic logics).

Equation S1 is a general signature subsuming many kinds of belief maintenance. It expresses the idea that an agent arrives at and maintains its beliefs by applying general background knowledge (an ontology) to specific situation data.

(S1) $${\frac{\text{Observation}\;\times \;\text{Ontology}}{\text{Belief}}}_{\text{BM}}\;\;\;{\frac{\text{Belief}\;\times \;\text{Ontology}}{\text{Belief}}}_{\text{BM}}$$

Signatures are read as follows: a cognitive state of the type below the line (here a Belief) is functionally dependent on the cognitive state above the line under some set of axioms or algorithms BM. The × operator may be understood declaratively (“together with”) or procedurally (“applied to”) whichever is more intuitive. Note that the second variant of the signature is recursive, so beliefs can propagate forward if the ontology warrants this.

### Canon 2: Raising goals

An agent needs to ensure continuity of its intentions and actions over time, mediated by the concept of a goal state.

A goal is a cognitive state that serves to coordinate an agent’s behavior even though circumstances may change and its decisions and plans need to be updated. Cognate concepts of goal include “desire” (as in intentional philosophy and in BDI theory); “drive” and “motivation” from classical psychology; “utility” from decision theory, “criteria” in multi-criteria decision models, and so on. The relationships between these terms are linguistically troublesome, but the concept has led to countless technical proposals in AI for representing and interpreting goal states in robots, planners, and other systems. The following signatures summarize the canon.

(S2) $${\frac{\text{Belief}\;\times \;\text{Ontology}}{\text{Goal}}}_{\text{G}}\;\;\;{\frac{\text{Goal}\;\times \;\text{Ontology}}{\text{Goal}}}_{\text{G}}$$

As before a cognitive state of the type below the line (here a Goal) is dependent on the agent’s knowledge and current cognitive state. In the first of the two signatures the state that leads to a goal may be a specific scenario such as *patient presents with severe and chronic pain* leading to two goals: *decide the most plausible cause* and *decide the most preferred treatment*. The second signature covers a case common in AI planning: goals lead to sub-goals. The goal *put out the fire* may entail sub-goals to decide how to *get* to the fire, the *strategy of attack*, the *equipment* needed and so forth, any of which may lead to further sub-goals.

The next four canons are core capabilities in goal-based decision-making.

### Canon 3: Generate options

An agent should apply specific knowledge of previously effective solutions when it can and use general knowledge to solve problems when specific knowledge is not available.

An agent may be able to identify multiple possible solutions so we refer to these as “candidates” in subsequent signatures, using the term to subsume other common terms like “decision option,” “problem solution,” etc.

(S3) $${\frac{\text{Goal}\;\times \;\text{Belief}\;\times \;\text{Ontology}}{\text{Candidate}}}_{\text{C}}$$

Signature Eq. S3 abstracts across “strong” problem solving methods, which draw on specific domain knowledge, and domain-general but “weak” methods like means-ends analysis and constraint solving (Laird et al., 1987). It also includes intermediate methods, such as heuristic classification, which maps between ontologies, as in *symptom* → *diseases* and *diseases* → *treatments* (Clancey, 1985) and explanation-based decision-making that depends upon building causal models for choosing between actions (Pennington and Hastie, 1993).

### Canon 4: Construct reasons

An agent should consider as many relevant lines of reasoning as is practical when establishing preferences over competing decision options.

There may be an indefinite number of reasons for accepting a hypothesis or selecting an action to achieve a goal. Reasons to believe in or to doubt hypotheses can be based on statistical evidence or logical justifications; reasons for choosing between alternative actions can be based on qualitative preferences or quantitative values. The following signature subsumes a range of strategies for constructing reasons for alternative options.

(S4) $${\frac{\text{Candidate}\;\times \;\text{Goal}\;\times \;\text{Belief}\;\times \;\text{Ontology}}{\text{Candidate}\;\times \;\text{Reason}}}_{\text{R}}$$

As Shanteau (1987) observed decision-making expertise is not only characterized by making good choices but also by meta-cognitive abilities like the ability to articulate the rationale for decisions. The signature provides for this meta-cognitive capability by making explicit the reasons for and against competing candidates.

### Canon 5: Aggregate reasons

When problem solving yields multiple candidate solutions an agent must establish an overall preference, taking account of all the reasons for each of the options.

Probabilistic updating is widely held to be the normatively correct way of establishing confidence in competing hypotheses, as is the expected utility extension for deciding about preferences over candidate actions. In many settings, however, it is impractical to estimate prior and conditional probabilities objectively, or to model costs and benefits on a single dimension. Simpler functions for determining overall preferences are helpful here, such as the Bentham rule (add up the *reasons pro* and *reasons con* and take the difference)^5^, or the equations of diffusion models (Ratcliff and McKoon, 2008). However there are many other aggregation functions which will deliver a preference ordering over the set of decision candidates. The signature below subsumes many specific functions that can establish the overall “merit” of a candidate.

(S5) $${\frac{\text{Goal}\;\times \;\text{Candidate}\;\times \;\text{Reason}}{\text{Candidate}\;\times \;\text{Merit}}}_{\text{Agg}}$$

One might assume that Merit must be a quantity, and aggregation a numerical algorithm. This is not necessarily the case. For example, we can describe preferences based on purely ordinal relations (A is preferred to B) and the preference determined on entirely logical grounds. Suppose, for example, that we have a reason R1 for preferring A, and a reason R2 for preferring B, but we also have some reason R3 which brings the veracity of R1 into doubt. All other things being equal we would prefer B to A. Informal preference rules, expected utility functions, argumentation systems and multi-criteria decision models are subsumed under this general signature.

### Canon 6: Commitment

If an agent can determine that its most preferred option will not change with further information then it should commit to that option. If there is missing information that, if known, would change the preference but the cost of acquiring that information is greater than the cost of taking the wrong decision then the agent can still be certain that its preference order will not change and a commitment can be made.

This canon of decision-making can be summarized by the following two variant signatures, covering the cases of *accepting a belief* (committing to one of a number of competing hypotheses) and *adopting a plan*.

(S6) $$\begin{array}{rcll} {\frac{\text{Candidate}\;\times \;\text{Merit}\;\times \;\text{Ontology}}{\text{Belief}}}_{\text{Accept}} & & & \\ {\frac{\text{Goal}\;\times \;\text{Candidate}\;\times \;\text{Merit}\;\times \;\text{Ontology}}{\text{Goal}\;\times \;\text{Plan}}}_{\text{Adopt}} & & & \end{array}$$

Whether a belief is acceptable or not is independent of the agent’s goals but the commitment to a plan is intimately bound up with the agent’s (prior) goals. The Goal is also retained below the line to indicate that the commitment only holds as long as the goal is extant.

The remaining set of signatures cover capabilities which may form part of a wider theory of cognitive systems, but are less relevant to the decision-making focus of the discussion and so are dealt with more briefly.

### Canon 7: Plan enactment

If an agent is committed to a plan that is necessary to achieve one or more of its goals, then enactment of the plan should be optimized with respect to the agent’s priorities and preferences.

Enacting a plan can be a simple sequential execution of the plan’s component steps, or involve flexible scheduling of the steps to accommodate changing circumstances. Enactment can be recursive, as execution of a step in the plan leads to new goals, which may require the current plan to be repaired, radically reconstructed, or abandoned. In all cases the effect of enactment is to update the current plan.

(S7) $${\frac{\text{Goal}\;\times \;\text{Belief}\;\times \;\text{Plan}\;\times \;\text{Ontology}}{\text{Goal}\;\times \;\text{Plan}}}_{\text{Enact}}$$

### Canon 8: Action

An action is defined as a plan step that cannot be decomposed into smaller elements. If the preconditions of a planned action are satisfied then it may be executed without further decision-making.

(S8) $${\frac{\text{Plan}\;\times \;\text{Precondition}}{\text{Action}}}_{\text{Execute}}$$

Preconditions include logical preconditions (e.g., a situation holds) and material preconditions (e.g., a physical resource is available).

### Canon 9: Monitoring

An agent should monitor the environment for important changes that may impact its goals and commitments and update its beliefs when necessary.

(S9) $${\frac{\text{Goal}\;\times \;\text{Observation}\;\times \;\text{Ontology}}{\text{Belief}}}_{\text{Mon}}$$

This can be viewed as a variant of the basic belief maintenance signature Eq. S1. If monitoring reveals that the preconditions of an intended action have ceased to hold (due to independent environmental changes or the effects of the agent’s actions for example) these actions should be postponed or discarded. If the new situation invalidates a past decision the agent should reconsider its options.

### Canon 10: Learning

An agent should update its knowledge as a result of experience.

There are many learning mechanisms, including associationist and statistical models in machine learning and cognitive neuroscience; case-based learning and rule induction in AI; reinforcement learning and “chunking” in cognitive psychology. Equation S10 represents learning as a generic process that updates its ontology by acquiring new scenario and task models.

(S10) $${\frac{\text{Belief}\;\times \;\text{Ontology}}{\text{Scenario}}}_{\text{CL}}\;\;\;{\frac{\text{Goal}\;\times \;\text{Belief}\;\times \;\text{Ontology}}{\text{Task}}}_{\text{IL}}$$

These signatures are only a starting point for describing learning in decision-making tasks; learning is a neglected topic in normative theory and behavioral studies of decision-making and we see this as a major challenge for general theories of DDM.

The 10 canons are offered as a first draft of a general framework for describing and discussing cognitive agents that can take decisions in the presence of uncertainty in dynamically changing situations. We do not claim that the set is complete (we are sure it isn’t) or that individual signatures cannot be improved (we know they can). At this point, however, we offer them as a basis for establishing an intuitive *lingua franca* for interdisciplinary discussions of decision-making theories, systems, and applications.

## Assessing the Canonical Theory

In this section we consider the adequacy of the framework in three representative applications: (1) understanding the gross structure of the human cognitive architecture; (2) collaborative decision-making by autonomous agents, and (3) designing decision support systems.

### Assessment 1: Dynamic decision-making and the human cognitive architecture

The canons discussed above are too general to make specific predictions about the detailed cognitive processes involved in human decision-making. They cannot therefore be seen as a theory of human decision-making as the lack of detail means they are not falsifiable in the Popperian sense (but see Lakatos, 1970; Cooper et al., 1996; Cooper, 2007). Our aims here are to show that the canons are nevertheless consistent with at least one large scale theory of the organization of cognitive processes, and that the framework provides a workable and useful approach to interpreting findings from cognitive neuropsychology and neuroscience.

The Soar cognitive architecture discussed in Section “Dynamic Decision Making” is one of several theories of the large scale organization of the human cognitive system that have been proposed and refined over the last half century (see also ACT-R: Anderson, 2007). These theories typically propose a set of functional *components* of the putative cognitive system, together with *interfaces* or mechanisms for interaction between those components, with the aim being to specify a system that is capable of supporting all aspects of human cognitive processing. The scope of these theories is therefore much broader than that of many theories in cognitive psychology, which typically focus on a single domain such as memory, attention, language comprehension, or choice.

**Figure 5:**
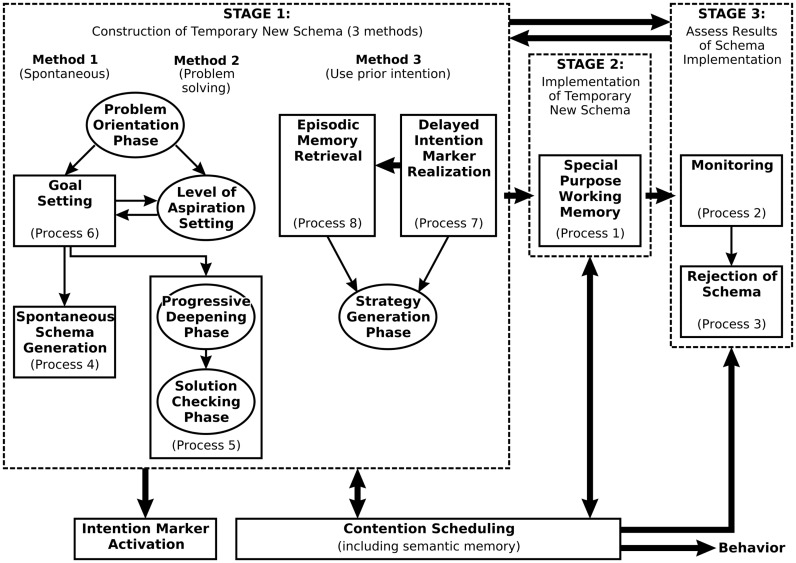
**The Contention Scheduling/Supervisory System model of Norman and Shallice (1986), as elaborated by Shallice and Burgess (1996)**.

While aspects of the canonical framework map on to processes or mechanisms within Soar, there is also a promising mapping onto the functional components of another cognitive architecture, namely the Contention Scheduling/Supervisory System (CS/SS) model of Norman and Shallice, 1986; see also Shallice and Burgess, 1996). The CS/SS model (Figure 5) has not been as well developed computationally as other cognitive architectures such as Soar or ACT-R. However, Glasspool (2005) proposed a mapping of the components of the CS/SS model to the domino architecture of Figure 3, and many of the model’s components can be understood as performing functions that correspond to the canons.

The CS/SS model draws a basic distinction between routine behavior (held to be controlled by learned schemas within the CS system) and non-routine behavior (held to be controlled through the construction and maintenance of temporary schemas by the SS). CS operationalizes canons 7 and 8: at the lowest level it maps plans to individual actions subject to their preconditions (Eq. S8), but CS is hierarchical, and at higher levels (even with routine behavior) it may map plans to sub-plans (Eq. S7).

In the initial version of the CS/SS model described by Norman and Shallice (1986) the output of the SS – temporary schemas for the control of behavior via CS – was specified but few details were given of the mechanisms by which such schemas might be generated. In response to concerns that it was homuncular, Shallice and Burgess (1996) elaborated the SS (see Figure 5), specifying eight processes, several of which were held to operate in different phases. Comparison of these processes with the canonical framework reveals many parallels, as well as possible limitations of both approaches.

Consider first belief maintenance (canon 1). The SS model does not explicitly include processes related to perceptual input or maintenance of declarative knowledge. There is thus nothing akin to canon 1, but an elaboration of the SS would clearly require such processes. Indeed, a more recent description of the SS model (see Shallice and Cooper, 2011, figure 12.27) includes such processes within Method 2 of the construction of temporary new schemas (cf. Figure 5).

Raising goals and problem solving are addressed explicitly within the SS model. Canon 2 is implemented via the Problem Orientation Phase, and specifically by process 6 (Goal Setting). Canon 3, by contrast, subsumes three routes within the SS model, corresponding to the three methods by which temporary schemas are generated. Temporary schemas can be related to “options” in the domino model but in this case the CS/SS model provides an account of decision-making that elaborates upon the canonical framework. This should not be surprising given that the canonical framework is intended as an abstraction over theories of decision-making.

Canons 4 and 5 (constructing and aggregating reasons) are incorporated in processes 4 and 5. In particular the Solution Checking Phase of the SS must evaluate candidate solutions and reject them if they have insufficient merit. However, the SS model lacks a detailed description of how this evaluation might occur. Canons 4 and 5 provide an abstract specification of the necessary processes.

The output of stage 1 and input to stage 2 is a commitment to a plan (canon 6).

Monitoring, as described by canon 9, corresponds directly to process 2 of stage 3 of the SS model. However, within the SS model the product of monitoring is not any belief, but a specific belief, namely that the current schema is not achieving the intended goal. This signals rejection of the current schema (process 3 of Figure 5), a meta-cognitive process that triggers another round of processing.

At least two forms of learning are supported by the CS/SS model: accretion of episodic memories, and proceduralization of frequently generated temporary schemas. The two variants of signature Eq. S10 are applicable, on the assumption that episodic memories are equated with scenarios and proceduralized schemas are equated with tasks. These assumptions are consistent with the usage of these terms and brings to the fore the potential value of describing a cognitive architecture within the canonical framework: it forces us to be explicit about cognitive constructs (schemas, episodic memories, etc.) and the relationships between them.

### Example: The Wisconsin Card Sorting Test

**Figure 6:**
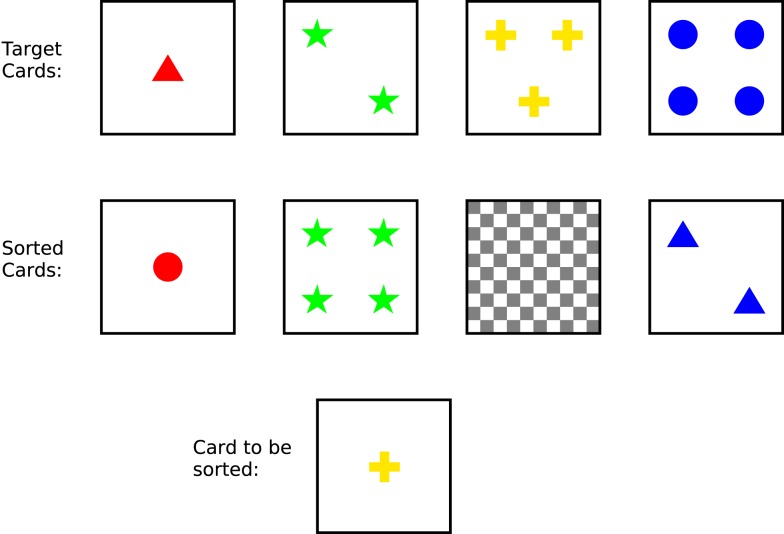
**The Wisconsin Card Sorting Test**. The four target cards are shown across the top row and four piles for sorted cards in the second row, the third of which is currently empty. The card to-be-sorted is at bottom. If the subject is sorting according to color or form, this card should be placed under the third target card, but if he/she is sorting according to number then it should be placed under the first target card.

We now consider the CS/SS model and assess whether details of *dynamic* cognitive processing on a laboratory task might be consistent with the canonical theory as well as the static cognitive architecture. In the Wisconsin Card Sorting Test (WCST) subjects sort a series of colored cards using feedback provided by the experimenter to deduce the appropriate sorting criterion. Each card shows one to four shapes (triangles, stars, crosses, or circles) printed in red, green, yellow, or blue. Four “target” cards are positioned at the top of the work surface (see Figure (6). A series of cards is then presented and the subject is required to place each one under one of the targets, after which the experimenter indicates whether the choice was correct. For example, the experimenter might first select color as the sorting criterion, giving positive feedback if the subject’s choice matches the color feature, but negative feedback otherwise. A complication is that after a series of correct choices the experimenter changes the sorting criterion without warning. The subject must therefore adapt his/her strategy throughout the task.

The WCST is not normally described as a decision-making task, though each trial requires a decision about where to place each successive card and the task as a whole has many features of DDM. In the standard presentation of the task, the cards to-be-sorted are in an ordered sequence and the first card shows one green triangle. Behavior on the task may be described as follows^6^:

Out the outset presenting a card to the subject places the subject in a situation for which no routine behavior exists. The SS is therefore invoked, raising the goal of sorting the card (Process 6 in Figure 5, or signatures Eqs S1 and S2 in the canonical theory). Problem solving (Eq. S3) can take at least two forms, depending on the subject’s strategy.

1. The first strategy simply yields four decision options corresponding to the locations under the four target cards. Suppose the to-be-sorted card shows one green diamond. A choice can be made on three lines of reasoning: (1) place the to-be-sorted card under target 1 because the shapes match; (2) place it under target 1 because the numbers match; (3) place it under target 2 because colors match. Aggregating the reasons (Eq. S5) there are now two arguments for placing the card under target 1 and one for placing under target 2, yielding a preference for target 1 so the subject places it under card 1 (Eq. S6).
2. A more sophisticated application of Eq. S3 yields an alternative strategy. Here the different sorting rules (sort by shape, sort by number, sort by color) can themselves be considered as decision options, with Eq. S4 being instantiated by arguments for/against each rule. Initially there will be no specific arguments for or against any rule so adoption of a specific rule (Eq. S5) would be random. Once a sorting rule has been selected, a further processing cycle of processing would be required to apply the selected sorting rule to the current card.

Are there any principled grounds for choosing between the simplistic and the sophisticated approach? Within the CS/SS model this will depend on the *aspiration setting*: if a specific solution is required (as may occur on trial 1) then the simplistic approach will suffice, but if a general solution is required (as subsequent trials demand) then the more sophisticated approach will be necessary.

After the subject adopts a plan to place the card (Eq. S7) and executes the placement (Eq. S8) the experimenter provides feedback. This feedback may trigger another round of processing. Effective use of feedback is a substantial source of individual variability on the task. More able, “attentive” or “energized” subjects may detect a learning opportunity (Eq. S1) and raise a goal to translate the feedback into another strategy (Eq. S2), with the options being the three sorting rules (Eq. S3: shape, number, or color); cycling through Eqs S4–S6 will yield beliefs relating to the veracity of each of these rules. In this way the canonical framework is able to describe meta-cognition in processing feedback as well as basic decision-making.

Processing is slightly different on the second trial because the arguments for placing a card under a particular target will have different merit. If feedback on the previous trial was positive (and is correctly assimilated) arguments for sorting by the rule(s) that matched on that trial will be stronger, while arguments for sorting by the rule(s) that did not match will be weaker. This potentially allows the cognitive system to be more discriminating about the options and select the correct target card, though additional trials and feedback cycles may be necessary to eliminate all but one sorting rule. However, once the subject has narrowed down the possible sorting rules to one it is possible to anticipate positive feedback. That is, in applying Eqs S7 and S8, the subject also establishes a belief that subsequent feedback from the experimenter will be positive. This makes monitoring (Eq. S9) a computationally simple process: if the observed feedback differs from the expected feedback then the subject may infer that the sorting rule applied on the current trial is incorrect. This will count as an argument against that sorting rule on the following trial. Failure to set up this expectation or to take account of violations of the expectation will result in the subject continuing to sort cards by a previously appropriate rule in the face of continued negative feedback (i.e., so-called perseverative errors).

Once the correct sorting rule has been determined it is necessary to maintain a record of this rule (presumably in working memory) across trials so that it may be used to support the argument for placing each to-be-sorted card under the matching target card. This is an instance of belief maintenance (Eq. S1), which as discussed above is not explicitly included within the CS/SS model of Shallice and Burgess (1996).

### The contention scheduling/supervisory system model as an instance of the canonical theory

The CS/SS model of the human cognitive architecture is not as well developed as some theoretical accounts of the human cognitive architecture, but it is unique within cognitive psychology in being grounded in a domain-general view of cognitive function and being supported by findings from neuropsychological studies^7^. For example, with respect to the WCST, subjects occasionally make “set loss” errors, where their behavior suggests that, after correctly inferring the sorting rule (as evidenced by a run of correctly sorted cards), they spontaneously forget the rule. Such errors are particularly common in neurological patients with lesions affecting the inferior medial prefrontal cortex (Stuss et al., 2000), a region Shallice et al. (2008) associate with “attentiveness.” Within the canonical framework, the subject’s difficulty here may be understood as concerning a particular aspect of belief maintenance (Eq. S1), but one that relates to retaining existing beliefs, rather than to deriving new beliefs, e.g., interpreting observations.

Perseverative errors, in which subjects fail to switch sorting rules in the presence of sustained negative feedback, are common in the behavior of patients with prefrontal lesions. Stuss et al. (2000) suggest that the perseverative errors of patients with lesions in right dorsolateral prefrontal cortex are due to monitoring failure. However avoiding perseverative errors also involves switching away from a previously reinforced rule, a process discussed in the psychological literature under the rubric of “set shifting” or “task setting“ which is frequently held to involve left dorsolateral prefrontal cortex. This process implements a form of Eq. S7 but the perseverative errors of different patient groups suggests there may be multiple underlying causes (Stuss et al., 2000), and as noted above, failures in monitoring (Eq. S9) may also result in such errors.

The functional components of the CS/SS model are supported by a great deal of evidence from cognitive psychology, cognitive neuropsychology, and cognitive neuroscience. Shallice (2006), for example, reviews a number of neuropsychological studies in which patient behavior may be interpreted as a specific impairment in “the production of one or more procedures for attaining a goal” (i.e., Eq. S3, the generation of candidate options). Other studies, also reviewed by Shallice (2006), imply that processes related to checking that on-going processing or behavior is working toward ones current goals may also be selectively impaired (see also Shallice and Cooper, 2011). Table 3 summarizes some of this evidence relating the processes of the CS/SS model to each of the signatures, drawing on further widely accepted views of the human cognitive architecture and its function in problem solving and decision-making.

**Table 3:** **Some relationships between the canonical functions and selected evidence from cognitive psychology and cognitive neuroscience**.

Signature	Summary
S1 (belief maintenance)	Beliefs may be supported by the environment (i.e., inferred from perceptual input) or inferred from long-term knowledge and other beliefs. Both must be actively maintained in working memory (e.g., by rehearsal)
S2 (raising goals)	Much behavior, with the possible exception of habitual behavior, can be understood as being purposive or goal-directed. In experimental psychology, high-level task goals are set by the experimenter, with subjects deriving lower-level goals for individual trials. Findings from experimental psychology and more generally indicate that goals provide local coherence of behavior
S3 (problem solving)	A variety of problem solving strategies or heuristics may be recruited to generate solutions for a given goal. This includes so-called “weak” methods which are general, knowledge-lean, heuristics such as hill-climbing and means-ends analysis, as well as knowledge-rich, task-specific strategies, acquired through experience
S4 (reasons for decisions)	Evolutionary arguments (e.g., Mercier and Sperber, 2011) suggest that argumentation is central to human decision-making. According to this view, generating arguments for or against propositions is an essential step in persuading others
S5 (aggregation)	One neuropsychological hypothesis is that aggregation of the merit of arguments is based on somatic markers – emotionally biased valences associated with decision options acquired through positive and negative experience (Damasio, 1994). Damasio relates the association of somatic markers with candidates to the amygdala and ventromedial prefrontal cortex
S6 (commitment)	Commitment to a single decision candidate is required by theories such as Damasio’s somatic marker hypothesis. In the specific context of selecting one word from a set, commitment has been related to the inferior frontal gyrus (Shallice and Cooper, 2011, Section 9.13)
S7 (plan enactment)	Plan enactment is most closely related to the function of task setting, held by many to be a function of left lateral prefrontal cortex (e.g., Shallice et al., 2008)
S8 (action)	The contention scheduling system provides an account of how intentions are mapped to actions, subject to available resources
S9 (monitoring)	A substantial body of evidence suggests that many cognitive processes create expectations that under normal operation are continuously monitored. Perceptual processes may also monitor the external environment for deviations from expected perceptual input. Shallice et al. (2008) relate monitoring to right dorsolateral prefrontal cortex, though an alternative view is that anterior cingulate cortex compares expectations with observations, generating an error signal when there is a mismatch
S10 (learning)	There are many forms of learning. One is learning to associate consequences with cognitive and motor actions. These consequences then become expectations which are used by monitoring. A second critical form is reinforcement learning, where positive or negative reward can increase or decrease the merit of a candidate in the context of a goal

### Assessment 2: Joint decision-making by autonomous agents

Understanding the foundations of autonomous operation of intelligent systems in complex, unpredictable environments is at the heart of AI. As discussed above it is a major focus of current research on software agents (e.g., Wooldridge, 2000; Fox et al., 2003; Poole and Mackworth, 2010). A major subfield of agent research is on *multi-agent* systems, in which autonomous agents interact to achieve common goals (Wooldridge, 2009). This field looks at models of how tasks can be shared between collaborating but individually autonomous agents, what forms of communication need to take place to achieve common objectives (such as informing, requesting, negotiating, persuading, and joint decision-making), and other cognitive functions.

**Figure 7:**
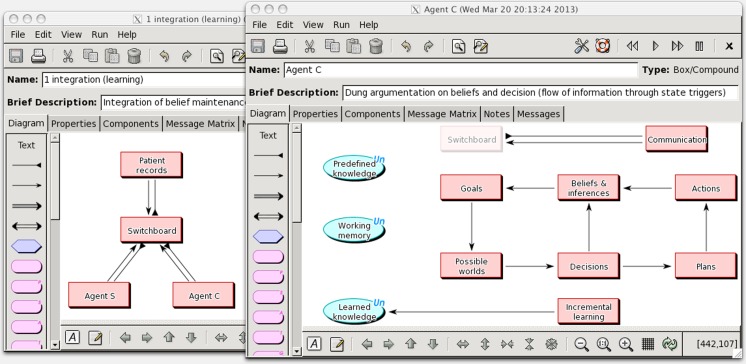
**Multi-agent network for cooperative decision-making (left), and information processing architecture for an autonomous agent (right)**.

We have carried out an initial assessment of the canonical theory by means of a computer simulation of a multi-agent decision-making task. The model has been built using the COGENT modeling tool, which is used to visualize the cognitive architectures of individual agents using an extended box-and-arrow notation, and implemented using rule-based and logic programing techniques. Figure 7 shows two views of an agent network in which three agents interact with each other in order to make a simple medical decision. They share information about a hypothetical patient with chest pain and two of them jointly arrive at a treatment decision. In this diagram ellipses represent various kinds of data repository and rectangles are “compound” modules that can contain lower-level information processing components (see Cooper and Fox, 1998; Cooper et al., 2002 for more detail). Arrows indicate flow of information between modules.

The left panel shows a network of agents in which the three outer rectangles represent agents which communicate with each other through a “switchboard.” A “patient records” agent provides information about patients with a specific medical problem (chest pain in this simulation). Agents C and S are the main actors in decision-making; agent C has cardiology knowledge and leads decision-making about the treatment of each patient, and agent S has specialist knowledge about safe drug use and can advise where there might be doubt.

The right panel shows the internal structure of agent C, which implements decision-making based on a version of the domino model extended with specialized stores for data and knowledge and processing modules which implement inter-agent communication and learning. Agent C, for example, has a set of data repositories which can be accessed by all processing modules: a *working memory* containing current cognitive states (beliefs, goals, plans etc); a *knowledge base* of domain facts, rules, and functions that are common to all agents (e.g., decision schemas, communication conventions), and specialist knowledge that is unique to each agent. Lastly, there is a knowledge base which contains a record of *past cases* and *learned knowledge* that can inform future decision-making. When this model is in operation working memory is constantly monitored by all the processing modules to determine whether any rules are applicable and, if so, the relevant cognitive state data are updated. These updates may lead to the conditions of other rules becoming instantiated, either within the same module (e.g., a new belief state propagates to further belief states) or another module (e.g., an updated belief state leads to an updated goal state).

### Example

This illustration^8^ has been selected purely to illustrate the operation of the decision model and is not intended to be medically realistic. Some operational detail is omitted for clarity.

#### Phase 1

Agent C receives information from the patient records agent saying that Mrs. Smith is an elderly patient who has complained of chest pain. From its knowledge about such problems agent C infers the possibility of a heart attack and from this the more specific possibility of a myocardial infarction (MI). Agent C’s knowledge indicates the need to “manage” the MI and a goal to achieve this is raised. Managing MI has in fact a number of aspects, including preventing blood clotting and pain and these are raised as sub-goals. Agent C consults its knowledge base and finds two drugs that might satisfy these goals: clopidogrel and aspirin. Both are effective for analgesia and prevention of blood clotting and easily available, and aspirin is also modestly priced. These arguments are weighed up leading to a simple conclusion that aspirin is preferred. However MI is a dangerous condition so the decision to prescribe aspirin is qualified as *provisional*, meaning that the agent will not act on the basis of this preference but will carry out further investigation and, depending on the results of this investigation, it may abandon the tentative decision in favor of an alternative.

#### Phase 2

**Figure 8:**
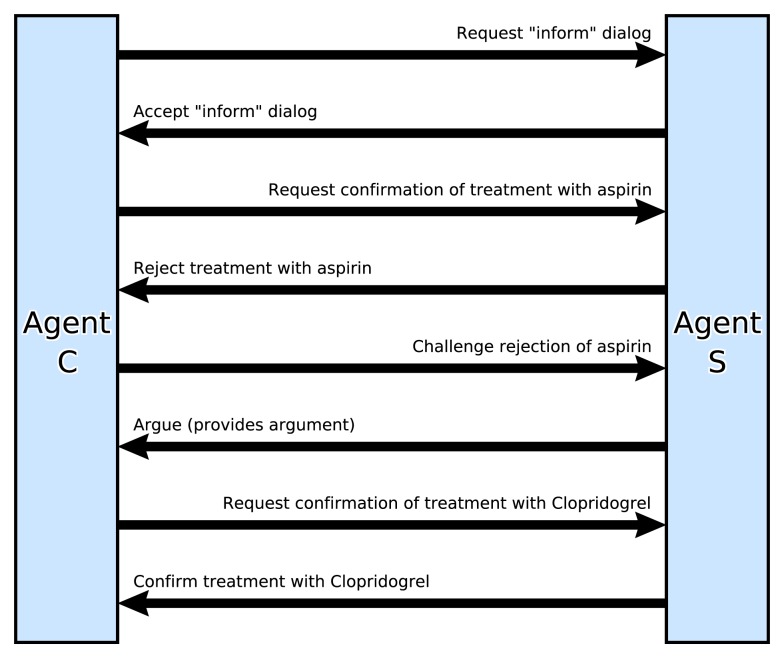
**Sequence diagram for some of the interactions between agent C and agent S during the joint decision-making simulation**.

The presence of a provisional decision triggers a rule in the goal processing module of agent C to raise a goal to consult another agent, S, that has specialist knowledge about the safety of drugs. C’s problem solver then retrieves a suitable interaction plan from its knowledge base. Communications are modeled using standard interaction “performatives” such as “inform,” “explain,” “query,” “request,” “instruct,” etc. Figure 8 summarizes the dialog that follows.

Agent C first sends a *request* to agent S to enter into an *inform* dialog, meaning that the two agents should follow a particular protocol for providing information. The request is accepted by S, so C sends a *request* for confirmation that aspirin is appropriate for Mrs. Smith. S then goes through its own decision process: raising a goal to decide on the best prevention of pain and clotting and generating a set of treatment options for Mrs. Smith. Its specialist knowledge of treatments for MI indicates aspirin, clopidogrel, and a further option: proton-pump inhibitors. S now proceeds to construct arguments for and against all three options. During this process it applies an argument schema that “If a treatment is proposed and is known to exacerbate a condition, and the patient has that condition, then this constitutes an argument against the proposed treatment.” Agent S has domain knowledge that aspirin exacerbates gastritis, and (after a *request* for information from the patient record agent) it finds that Mrs. Smith has gastritis so this yields an argument against prescribing aspirin. S *informs* C that clopidogrel is therefore preferred.

#### Phase 3

Agent C raises a goal to understand the rationale for the Agent A’s advice. One way of doing this is to “challenge” the advice in order to elicit the reasons for the recommendation. Agent S reflects on its rationale and provides an explanation as a set of arguments in an *inform* message. This leads to Agent C adding another argument against aspirin to working memory and clopidogrel is now the preferred option for Agent C as well.

#### Phase 4

As a final part of this experiment we implemented a simple learning mechanism for acquiring new knowledge which can be used in future decisions based on a record of the whole episode. An episodic record of the decision includes the goal that was active and the set of beliefs that held when a decision was committed. Another learning mode was also demonstrated by recording how frequently particular decisions are taken in particular contexts, which can be used to weight options in future decisions.

### The agent architecture as an instance of the canonical theory

The agent architecture owes much to the domino framework so there is naturally a good mapping between this implementation and the canonical framework (Table 4). Phase 1 traverses Eqs S1–S6, and phase 2 traverses Eqs S2–S8. Communication is modeled by means of goal-based dialogs represented as plans (Eq. S6), and individual communication acts are instances of the action signature Eq. S8.

**Table 4:** **The relation between the canon signatures and functions which are implemented in the multi-agent decision-making scenario**.

Signature	Summary
S1 (belief maintenance)	Any rule in the agent model can make inferences by applying knowledge to the current working memory state and add, delete or replace information in the working memory. Every item of data in working memory is tagged with the grounds for believing it (e.g., the goal and assumptions which justify it). It uses this to maintain a consistent overall belief state
S2 (raising goals)	Goals are a form of belief which are used to determine which knowledge and rules are potentially in play at any moment
S3 (problem solving)	Any kind of problem solving technique can be implemented in the COGENT programing system, with the solution then added to working memory
S4 (reasons for decisions)	A form of argumentation based on defeasible logic is used to generate and maintain arguments for competing solutions as the working memory belief state changes
S5 (aggregation)	In the multi-agent decision-making scenario a simple improper linear aggregation function is implemented (adding up pros and cons) though other aggregation functions can be implemented
S6 (commitment)	The multi-agent scenario includes two kinds of commitment, provisional (reversible), and firm (irreversible)
S7 (plan enactment)	Dialog plans are simple lists of communication actions that are executed in sequence but can be interrupted if a communication is received from another agent
S8 (action)	The main kinds of actions that are included in this demonstration are standard communication performatives from speech act theory and agent communication languages
S9 (monitoring)	The whole domino system is a kind of “monitor” in that every computational component can respond to any update to the working memory state at any time
S10 (learning)	Two simple learning mechanisms have been implemented. These monitor the working memory and when a decision process terminates these mechanisms (1) add rules to the agent’s episodic knowledge and (2) update frequency counters which can be used to update the agent’s confidence in competing decision options

The signatures specify general constraints on the inputs and outputs of each information processing component of the agent architecture. At this abstract level each component is a black box and may be implemented in any number of ways: as a conventional algorithm, a set of production rules, a logic program, in hardware, or in some other way. In the COGENT implementation the signatures are translated into eight sets of specialized production rules, one set associated with each component.

### Assessment 3: Designing decision systems

Our research was originally motivated by a wish to understand cognitive processes that underpin human judgment and to apply this understanding by developing a technology for designing decision-making systems for use in dynamic, complex, and safety critical settings (Fox and Das, 2000). Taking medicine as a concrete focus we established three engineering requirements.

### Scope

The technology should be able to model any type of decision, represented by the corpus of decisions common in clinical practice (e.g., hazard detection, risk assessment, test selection, referral, diagnosis and treatment decisions, and many others). The framework should also cover the full lifecycle of decision-making from the point where the need for a decision is established to the point where a choice can be made. A general *symbolic decision procedure*^9^ was developed for this purpose (Fox et al., 1990; Fox and Krause, 1991; Huang et al., 1993) which later evolved into the domino model.

### Realism

Applications must cope with the constant change and high-levels of uncertainty typical of real world environments, where quantitative decision models are impractical (e.g., due to lack of data) and which naturalistic decision models seek to address. A key proposal was a preference and choice model based on logical argumentation (Fox et al., 1993; Krause et al., 1995) which has features in common with reason-based choice in cognitive psychology (e.g., Shafir et al., 1993), formal argumentation for decision-making in AI (e.g., Amgoud and Prade, 2009) and theories of argumentation in social and evolutionary psychology (e.g., Mercier and Sperber, 2011).

### Implementability

The practical development of decision support services requires an expressive implementation language for modeling and implementing decisions and other tasks. The symbolic decision procedure and argumentation model proved to be an effective foundation for a practical decision modeling language (Das et al., 1997; Fox and Das, 2000), the most developed version of which is a published standard (Sutton and Fox, 2003). *PROforma*^10^ has proved to be capable of modeling a wide range of decision processes in a way that clinicians find natural to understand and use. It has been used to deploy many clinical applications which are in routine use (e.g., the NHS Direct triaging service in the UK^11^; support for decision-making by multidisciplinary teams, Patkar et al., 2012).

**Figure 9:**
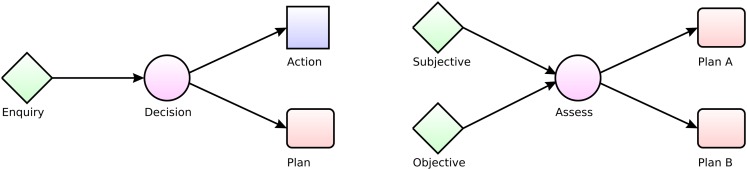
**Simple *PROforma* task networks (The Tallis decision support development software can be downloaded from www.cossac.org/tallis for research use)**.

PRO*forma* reifies the logical processes of the domino into a *task model*. It is a knowledge representation language (for modeling expertise) and a programing language (for implementing decision support systems and autonomous agents). A typical PRO*forma* model is a network of decisions, plans and other tasks which can be enacted in a predefined sequence, or concurrently, or in response to circumstances. Two simple example networks are shown in Figure 9.

The first example starts with an “enquiry” (any data acquisition process, shown as a diamond). This may acquire data from many sources (e.g., database, a sensor or other device or querying a human user or another agent). The decision (circle) that follows the enquiry can be taken only when the enquiry has completed, which is specified by the connecting arrow. The decision applies relevant knowledge to interpret the data that has been acquired up to this point by constructing and assessing arguments for and against the various decision options. One option here is a simple action (e.g., send the patient home) while the other is a plan (e.g., a course of treatment).

The second example captures a decision-making pattern called SOAP (for “Subjective”; “Objective”; “Assessment”; “Plan”) which is a mnemonic familiar to clinicians that refers to the routine process of taking a patient history, deciding what to do, and then doing it. There are two enquiries in this example; one acquires information about the patient’s subjective complaint and experience while the other captures objective data such as the patient’s height, weight, and blood pressure. It does not matter in what order the data are acquired but the *Assess* decision may not be taken until both enquiry tasks have been done.

### *PROforma* as an instance of the canonical theory

As shown in Table 5 the *PROforma* language instantiates signatures Eqs S1–S8 in the canonical framework, though different interpreters for the language implement some details differently.

**Table 5:** **The relation between the canon signatures and task representations in the *PROforma* modeling language**.

Signature	Summary
S1 (belief maintenance)	Beliefs in *PROforma* are data derived from the external environment (e.g., “age = 54”) or indirectly by inference or decision-making (“diagnosis = ulcer”) and also “meta-data” (e.g., “diagnosis = completed”). If the data change then beliefs can be automatically updated, e.g., order of preference over a set of options in a decision that is currently in progress
S2 (raising goals)	A *PROforma* goal is a logical combination (and/or/not) of situation descriptions which do not currently hold. A goal can be raised by any kind of task; the task will be terminated if the goal descriptions become true
S3 (problem solving)	Current *PROforma* interpreters are limited to retrieving a set of options from a knowledge base
S4 (reasons for decisions)	Reasons in *PROforma* are *logical arguments* that represent *evidential arguments* in deciding between hypotheses and *preference arguments* when deciding between actions
S5 (aggregation)	A decision assesses all the argument for and against each option to determine their net overall force, and establish an order of preference over the options. The prior confidence and strength of arguments can be taken into account in the aggregation process
S6 (commitment)	Each option in a *PROforma* decision may include a rule which defines the conditions in which the option can be “recommended” when the application is supporting a third party decision, or automatically “committed” if the system is configured to operate autonomously
S7 (plan enactment)	A *PROforma* plan is a network of tasks, in which the scheduling of decisions and other tasks can be predefined or determined dynamically
S8 (action)	When a *PROforma* action is scheduled for execution it first checks any preconditions (such as beliefs or goals being true, resources being available)
S9 (monitoring)	There is no specific support for monitoring. However continuous monitoring can be implemented using general language features
S10 (learning)	The *PROforma* language standard does not currently support learning

## Summary and Discussion

We have briefly examined traditional perspectives on decision-making, including decision theory (prescriptive models grounded in rational axioms); decision science (descriptive, empirically grounded theories of decision-making), and decision engineering (techniques for supporting human decision-makers and developing autonomous decision agents). The central motivation for developing a canonical theory is to provide a *lingua franca* for discussions between researchers, and with practitioners, based on a common set of intuitive but well-defined concepts and processes. This project will be successful if members of community A find the framework sufficiently versatile and clear for describing their view of decision-making to members of community B and vice versa, and if productive conversations ensue.

The canonical framework developed here does not fit squarely into any one of the traditional paradigms. The canons are neither normative nor descriptive; we have used the term “requisite” elsewhere (Fox, 1981)^12^. The canons say that any general decision procedure must address certain functional requirements in some way (maintaining beliefs, raising goals, making commitments, and so forth). However we are not imposing any particular way in which the canons must be implemented. That is, the canons are framed at the highest of Marr’s levels (Marr, 1982): they specify what function should be computed but make no commitment to the algorithms involved or the representation of information over which they operate. This is in contrast with a normative theory like expected utility theory that commits the implementer to update belief in a way that is constrained by the probability axioms and measures of value must satisfy “rational” axioms like transitivity and so forth. Our only claim is that any proposal for a specific theory of DDM must implement some or all of the canons in some way.

A clear limitation of our program, consequently, is that the canonical theory does not address the particular concerns of each decision research community in detail. Psychological mechanisms are insufficiently specified in the canonical signatures to make predictions about how human decision-makers actually behave or how human performance differs from prescriptive norms. Nor can the canons be claimed to be axioms of rational inference, such as those offered by statistical decision theory or mathematical logic. Lastly the canonical form does not offer tools for designing practical applications. Nevertheless a canonical framework may have benefits for specialist researchers in all three traditions in that the general canons can be instantiated for particular purposes by specific procedures or mechanisms. We close with a short discussion of some of the benefits that the framework may offer to theorists, scientists, and engineers.

### Contributions to decision theory

1. General canons of cognition help to promote discussion between communities with different theoretical commitments. The belief maintenance canon Eq. S1 for example can be instantiated by probabilistic inference, fuzzy logic, default reasoning, and so on. These are often seen as competitors but in our view it would be more helpful to see them as alternative ways of modeling uncertain reasoning to address different requirements and constraints.
2. The canons offer a broader context within which to investigate formal theories of decision-making than is usual. Classical expected utility theory, for example, “can give no scientific advice” about when a decision is needed or what is relevant to framing it (Lindley, 1985). This seriously limits the scope of current theory and the canonical framework suggests a number of ways in which normative models could be extended.

### Contributions to decision science

1. Canonical forms can be used to provide functional explanations of behavioral, clinical, or neurological data obtained in decision-making tasks and to map between neuro-anatomical organization and cognitive-level functions (Shallice and Cooper, 2011).
2. The theory bridges “folk” psychology, common sense reasoning, agent theories in AI and philosophical theories of mind, and potentially engages with the vocabulary of the humanities and everyday discourse.
3. The canonical theory provides a framework that may help to resolve debates which arise from different assumptions about methodology. A current dispute in cognitive science, for example, concerns whether psychological theories are constrained by neuroscience data and vice versa. Coltheart (2006) has argued that a *true psychological theory* exists at such a level of abstraction that data about the neurological implementation of cognition cannot in principle confirm or refute the theory. The abstract cognitive theory that Coltheart seeks is at the canonical level – it can inform psychological theory at a functional level but need not confront implementation details which are particular to human cognition.

### Contributions to decision engineering

1. The abstract signatures are insufficiently specified to be directly computable but there are clearly many specific algorithms that will take, as input, data of the types specified “above the line” and generate, as output, data of the types specified “below the line”^13^. Rather than just making the theory too vague to be useful this has the practical implication that we could design and implement decision-making systems by assembling them out of standard components which comply with the signatures without knowledge of internal mechanisms.
2. Responsibility for practical decision-making is often distributed across professional teams and in the future such teams will be increasingly supported by automated services. In an emergency management system for example distinct sub-systems will be responsible for capturing data while others will be required to integrate data from multiple sources and “judge its sense, meaning, relevance, and reliability; decide what the options for action are and make effective decisions” (Carver and Turoff, 2007, p. 34). Faced with this complexity designers will wish to engineer systems using standard modules for decision-making, planning, communication etc. and a canonical model offers a way of specifying and linking such modules.

## Conclusion

In this paper we have considered the whole cycle of DDM: recognizing and framing a problem in light of current beliefs; clarifying and prioritizing goals; generating options that would achieve current goals; evaluating preferences over the options; and aggregating preferences to select the best. We have not sought to develop any new theory related to the specifics of any one of these capabilities. Rather, our approach has been to develop an over-arching framework that subsumes specific theories of these individual subprocesses and understand how they are related to each other.

In our view theoretical understanding of the processes involved in human DDM is advancing, but in a somewhat chaotic way in which many competing research traditions, theoretical concepts, and engineering techniques vie for pre-eminence. We hope that our discussion has demonstrated that there is potential for establishing a cross-disciplinary framework that promotes constructive discussion between communities, leading to collaboration, and even synergy rather than competition.

## Conflict of Interest Statement

The authors declare that the research was conducted in the absence of any commercial or financial relationships that could be construed as a potential conflict of interest.

## Footnotes

^1^Soar was the first computationally well-specified cognitive architecture (see Newell, 1990). While Soar continues to be developed (e.g., Laird, 2012), discussions of cognitive architecture are now generally dominated by Anderson’s (2007) ACT-R theory. Our focus on Soar is based on Soar’s decision process and what Newell (1990) referred to as the *Problem Space Computational Model* (cf. Figure 2). This provides an intermediate level at which behavior may be described. This level of analysis is not considered within ACT-R.

^2^The Institute of Medicine’s famous report *To err is human* (Kohn et al., 2000) put patient safety at the top of the international health care agenda in 1999 and its ramifications are still being felt.

^3^We make two important caveats. First, we have adopted a particular vocabulary of cognitive states, such as “belief,” “goal,” “argument,” “plan” etc. These terms are common parlance, but are often also used with a technical interpretation that differs from community to community. It would in fact be preferable to avoid these overloaded terms (perhaps calling the different types of state “beta,” “gamma” “alpha,” “pi,” or some such) but we will persist with this more familiar terminology to make the presentation easier to understand. Readers should, however, be wary of interpreting the terms colloquially and keep the canonical meaning in mind. Second, we have adopted a language which distinguishes between different types of information (beliefs, ontologies, goals, etc.) in order to specify the canonical functions that we believe a decision-maker of the sophistication of a human agent needs to implement, but this does not imply that the internal implementation of the function must be symbolic or propositional. Moreover, use of a notation for cognitive states should not be taken to imply that we are speaking exclusively about agents that have explicit mental states. The use of signatures is agnostic about any underlying implementation provided that implementation is consistent with the constraints defined by the signatures.

^4^At the informal end of our field of interest are the humanities, subsuming philosophy and literature, social commentary and political theory, and so on. Even here some notion of belief maintenance is needed, though these areas are primarily served only by everyday language rather than technical frameworks. In fact natural languages are of course hugely expressive tools, and articulate many distinctions and subtle nuances around the idea of belief such as *possibility*, *plausibility*, *conviction*, *assumption*, *expectation*, *suspicion*, and *doubt*. It can be argued that such concepts also deserve a place in any discussion of decision-making (e.g., Fox, 2011) and deal fundamentally with the concept of belief maintenance as well. Such attitudes are frequently treated as merely part of our folk psychology and theoretically uninteresting. In our view they capture real distinctions which are important in all practical thinking and social interaction.

^5^technically a linear function with equal weights

^6^This decomposition of WCST processes is based closely on a computational model of WCST performance described by Glasspool and Cooper (2002).

^7^Other cognitive architectures such as Soar (Newell, 1990) or ACT-R (Anderson, 2007) are grounded in specific areas of cognition (e.g., problem solving or associative memory) and view cognitive processing as equivalent to the operation of a production system.

^8^The illustration is based on an example developed by Sanjay Mogul.

^9^“A symbolic decision procedure can be characterized as an explicit representation of the knowledge required to define, organize and make a decision, and … a logical abstraction from the qualitative and quantitative knowledge that is required for any specific application. A SDP may include a specification of when and how the procedure is to be executed” (Fox and Krause, 1991, p. 106).

^10^A contraction of *process* and *formalization*.

^11^http://www.nhsdirect.nhs.uk/CheckSymptoms.aspx

^12^The term derives from Ashby’s Law of Requisite Variety (Ashby, 1958), which concerns the regulation of the behavior of a system, R. The law states that “R’s capacity as a regulator cannot exceed its capacity as a channel for variety” (Ashby, 1958, p. 86). The law is “requisite” in the sense that it states a requirement on the variety possible within the system R.

^13^Each signature can be thought of as a pair of colored sockets into which standard cognitive components can be plugged if and only if colors of the plugs match colors of the sockets.
